# The role of brevican in glioma: promoting tumor cell motility *in vitro* and *in vivo*

**DOI:** 10.1186/1471-2407-12-607

**Published:** 2012-12-19

**Authors:** Renquan Lu, Chengsheng Wu, Lin Guo, Yingchao Liu, Wei Mo, Huijie Wang, Jianbo Ding, Eric T Wong, Min Yu

**Affiliations:** 1 Department of Biochemistry and Molecular Biology and the Key Laboratory of Molecular Medicine, Ministry of Education, Shanghai 200032, China; 2 Department of Clinical Laboratory, Fudan University Shanghai Cancer Center, Shanghai, China; 3 Department of Neurosurgery, Provincial hospital affiliated to Shandong University, Jinan, China; 4 Department of Clinical Oncology, Fudan University Shanghai Cancer Center, Shanghai, China; 5 Gamma Knife Center, Huashan Hospital, Shanghai, China; 6 Brain Tumor Center and Neuro-Oncology Unit, Department of Neurology, Beth Israel Deaconess Medical Center, Harvard Medical School, Boston, MA, USA

**Keywords:** Brevican, Glioma, Astrocytoma, Motility, Tumorigenicity

## Abstract

**Background:**

Malignant glioma is a common primary tumor of the central nervous system. Brevican, an abundant extracellular matrix component in the adult brain, plays a critical role in the process of glioma. The mechanisms for the highly invasive behavior of gliomas are still poorly understood. The aim of this study was to examine whether brevican is a predictor of glioma and its roles in glioma cell motility.

**Methods:**

In this study, immunohistochemistry staining for brevican expression was performed in malignant gliomas and benign controls. We also explored the effects of brevican on cell adhesion and migration in brevican-overexpressed cells. Knockdown of brevican expression was achieved by stable transfection of U251 cells transduced with a construct encoding a short hairpin DNA directed against the brevican gene, which correspondingly, down-regulated the proliferation, invasion and spread of brevican-expressing cells. Moreover, the role of brevican in the growth and progression of glioma was demonstrated by *in vivo* studies.

**Results:**

Our results provide evidence for the molecular and cellular mechanisms that may underlie the motility-promoting role of brevican in the progression of glioma. The role of brevican as a target for immunotherapy might be taken into consideration in future studies.

**Conclusions:**

This study suggests that expression of brevican is associated with glioma cell adhesion, motility and tumor growth, and also is related to glioma cell differentiation, therefore it may be a marker for malignance degree of glioma

## Background

Malignant glioma, the most common primary tumor in the central nervous system (CNS) with an almost invariably rapid and lethal outcome, is characterized by a distinctive ability to invade the surrounding tissue [1]. Tumor cell invasion is a particular problem at the time of recurrence after the failure of anti-angiogenesis treatment, resulting in neurological deficits and eventual patient demise [2,3]. Yet, at this time, no drug treatment is available that can interfere with the invasiveness of malignant gliomas.

Brevican is one of the most abundant proteoglycans (PGs) in the postnatal brain and is the smallest core protein among the lectican family [4-6]; its gene is located on chromosome 1q31 and includes 14 exons [7]. Recent studies have shown that brevican expression is restricted within the CNS, including the brain and spinal cord, but is absent in extracranial organs, such as the heart, muscle, liver, kidney, lung, thymus and spleen [8]. Interesting, malignant gliomas exhibit unique brevican isoforms, and brevican is critical for its proinvasive role in glioma [9].

Brevican expression is induced in intracranial grafts of invasive glioma cell lines [10]. Evidence has shown that the coding sequence of the brevican gene in glioma is the same as the brevican gene found in the cortex of the normal brain. Therefore, the regulation of expression of brevican in glioma is not caused by mutation [7]. To date, our understanding of the regulatory mechanisms of brevican functions and the involvement of PGs in cancer is limited. In our previous mass spectrometry screening in the cerebrospinal fluid (CSF), we found brevican was overexpressed in glioma patients. In this paper, the implication of brevican in cancer development and progression is discussed. Our results demonstrated a motogenic role for brevican and suggested that brevican was a key enhancing factor in the progression of glioma. Targeting brevican might offer a novel and promising approach to cancer immunotherapy by engaging the tumor microenvironment.

## Methods

### Tumor tissues of glioma and the control group

Formalin-fixed paraffin-embedded tissues from 60 patients with glioma of the astrocytoma cell types (grades I, II, III and IV, *n* = 15 for each) and 40 patients with non-glioma CNS tumors, including meningioma (*n* = 20) and pituitary adenoma (*n* = 20), were used in this study. All subjects (53 male, 47 female; aged 13–68 years) were retrieved from the archived cases at the Department of Pathology, Fudan University, Shanghai Medical School (Shanghai, China). The clinicopathological characteristics of the 60 glioma patients are shown in Table 1. All of these patients gave their informed consent for this research. This study was approved by the Institute Research Committee at Fudan University, Shanghai Medical School.

**Table 1:** The characteristics of 60 patients with malignant glioma

**Characteristics**	**Number of patients (N = 60; %)**
Age (years)	
≤50	32 (53.3)
>50	28 (46.7)
Gender	
Male	30 (50.0)
Female	30 (50.0)
Histological grade	
I	15 (25.0)
II	15 (25.0)
III	15 (25.0)
IV	15 (25.0)
Degree of differentiation	
Well (hair cell type-, oligodendrocyte type- astrocytoma)	30 (50.0)
Poorly (anaplastic astrocytoma, glioblastoma)	30 (50.0)

### Cell lines

The human glioma U251MG and U87 cell lines, and the non-glioma cell line 293T were obtained from the American Type Culture Collection (Manassas, VA). The cells were grown in DMEM medium (Invitrogen, Grand Island, NY) supplemented with 10% fetal bovine serum, 50 units/mL penicillin, and 50 μg/mL streptomycin in a humidified atmosphere with 5% CO_2_ at 37°C.

### Construction of recombinant plasmids and production of anti-brevican antibodies

The pIRES-hrGFP-brevican plasmid containing the full sequence of brevican was provided by a Department of Neurology laboratory at the Beth Israel Deaconess Medical Center. The brevican fragment was subcloned into the pMX-puro(+) vector (Invitrogen) to yield pMX-brevican, which was then transfected into 293T, U251 and U87 cells using Fusion 6® (Roche, Mannheim, Germany). In addition, the DNA sequence for the N-terminal domain (aa 22–104) of brevican was amplified using the primers 5’-ACGGATCCGCAGATGTTCTGGAAGGAGACA-3’ (P1) and 5’-CCGCTCGAGGTAGGCCTCGTTCACCTTGAC- 3’ (P2). The brevican N-terminus was also subcloned into the PGEX-4T-1 expression vector (Invitrogen), and brevican recombinant protein was obtained successfully. The anti-brevican antibody *B5* was obtained using immunized New Zealand rabbits, performed as previously described [11].

### Immunohistochemical (IHC) staining

The paraffin sections were dewaxed and hydrated, followed by antigen repairing for 20 min. Rabbit anti-brevican antibody (B5) was then added at 4°C overnight, and horseradish peroxidase labeled anti-rabbit IgG at 37°C was incubated for 1 h. Then 0.05% DAB was added for 5 min, hematoxylin for 1 min, and eosin for 2 min. The IHC sections were stained by hematoxylin and eosin (HE), and scanned under microscopy. The positive index (PI) was calculated using the following formulation: *PI* = *i* × *p,* where *i* is intensity of staining (0 for negative, blue; 1 for weakly-positive, light yellow; 2 for medium positive, yellow; 3 for strong positive, brown), and *p* is positive percentage of staining (1 for *≤*10%; 2 for 11%-50%; 3 for 51%-75%; 4 for *>*75%) [12]. The *PI* of glioma specimens was compared with that of the control tumors.

### Brevican knockdown

Knockdown of brevican expression was achieved using recombinant plasmids containing short hairpin DNA (shDNA), which were constructed by cloning the respective shDNA into the pSuper-puro vector (Invitrogen). The candidate sequences of the shDNAs were as follows: shDNA 1, 5’GATCCCCGGTGAACGAGGCCTACCGGTTCAAGAGACCGGTAGGCCTC GTTCACCTTTTTGGAAA3’, shDNA 2, 5’GATCCCCGTTATGCTGAAGACCTAAATTC AAGAGATTTAGGTCTTCAGCATAACTTTTTGGAAA3’, shDNA 3, 5’GATCCCCGGAG GAAGAAGAGAAATATTTCAAGAGAATATTTCTCTTCTTCCTCCTTTTTGGAAA3’. A mock plasmid was constructed using the scrambled shDNA sequence 5’-GATCCCCGCTCCTAGAATTTGAAACATTCAAGAGATGTTTCAAATTCTAGGAGCTTTTTGGAAA-3’. Stably transduced U251 cells that overexpress brevican were transfected with these plasmids for 24 h and then treated with 1.0 μg/mL puromycin. The remaining cells were cultured with 0.5 μg/mL puromycin until cell colonies formed, and Western blots were used to test for brevican levels.

### Cell adhesion and migration assays

The stably transduced cells were resuspended in culture medium. A total of 50,000 suspended cells were added to a 96-well plate coated with human fibronectin (20 μg/mL; Solarbio, Beijing) and poly-L-lysine (50 μg/mL; Sigma, St. Louis, MO). After an 1 h incubation, the plates were washed with PBS, fixed with 4% paraformaldehyde, and the 570 nm absorbance was quantified after crystal violet staining. For the migration assay, cells diluted in serum-free culture medium were plated onto 24-well plates preloaded with Transwell inserts (8 μm pore size; Costar, Cambridge, MA) at 50,000 cells per well in 100 μl culture medium and allowed to migrate for 10 h. Subsequently, the cells that had migrated to the underside were stained and counted using microscopy. All experiments were repeated at least three times.

### Cell proliferation, invasion and wound healing assays

The transduced cells were plated on 24-well plates (1000 cells/well) and the cell colonies were counted at day 12 to measure the cell proliferation rate. For cell invasion experiments, Matrigel was plated inside Transwell culture inserts for 5 h at 37°C before cells were plated onto the inserts (50,000 cells/well). After 16 h, the cells that migrated to the underside were stained and counted.

For the cell wound healing assays, a cell scraper was used to create scratch wound on a dish with cells grown to 80% confluence. The distances of the wounds were then measured using a microscope at 0, 24 and 48 h. Cell motility was evaluated using the following formula: cell motility = (distance_24h or 48h_ – distance_0h_)/ distance_0h_.

### Cytoimmunofluoresence staining

Cells were grown on a slide chamber for 24 h, fixed for 10 min in a cold mixture of methanol and acetone, and blocked with PBST containing 0.5% bovine serum albumin for 2 h. Cells were incubated with anti-brevican polyclonal antibody B5 (1: 100) overnight at 4°C, followed by incubation with CF^TM^ 488A labeled goat anti-rabbit secondary antibodies (Biotium, Hayward, CA) for 30 min at 37°C. The cells were then observed under fluorescence microscopy.

### Western blot

Whole cell lysates were used for immunoblotting as described previously [13]. Enhanced chemiluminescence detection was performed according to the manufacturer’s instructions with an ECL kit (Thermo Scientific, Rockford, IL).

### Tumorigenicity analysis

To validate the effects observed with brevican knockdown *in vitro*, BALB/c nude mice were injected subcutaneously into the right flank with either the 5×10^6^ U251 transduced cells, transduced U251/brevican-shDNA mock, or transduced U251/ brevican-shDNA 2. BALB/c nude mice (five mice per group) were purchased from the National Rodent Laboratory Animal Resources (Shanghai). Tumor measurements were made every 4 days and tumor sizes were calculated using the formula V= 0.5*a*’ × *b*’^2^, where *a*’ and *b*’ were the long and short diameters of the tumor. In addition, 2×10^5^ cells were intracranially injected into the right thalamus of BALB/c nude mice using a 10 μl syringe. A total volume of 8 μl cell suspension was injected at 2.5 mm anterior to the bregma and 2.0 mm lateral to the midline into three mice for each group. Magnetic resonance tomography (MRT) was used for comparing the orthotopic tumor growth in the nude mice.

### Statistical analysis

A one-way ANOVA was performed using SPSS 13.0 software (SPSS Inc., Chicago, IL). The results were expressed as the means ± SD, and a *P* value < 0.05 was considered to be statistically significant.

## Results

### Brevican is differentially expressed in glioma and benign brain tumors

**Figure 1:**
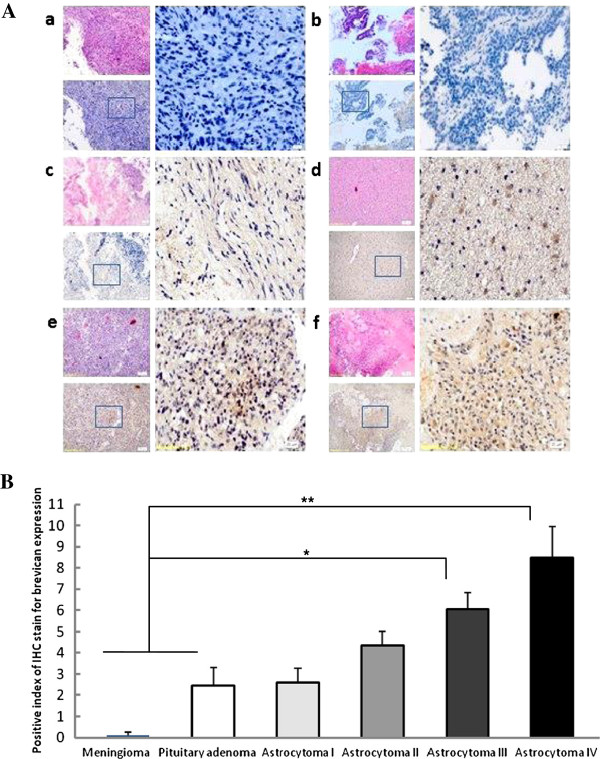
**Brevican expression levels were detected by immunohistochemistry staining using an anti-brevican antibody.** (**A**) The staining results were observed by microscopy. Meningioma was negative (*a*, blue*; n* = 20), pituitary adenoma was weakly-positive (*b*, light yellow*; n* = 20) and malignant glioma sections (astrocytoma grades I-IV, *n* = 15 for each) were positive (*c- f*, yellow to brown; respectively). Hematoxylin was used for nuclear counterstaining (blue). *Left*: 100×, HE staining (*above*), IHC (*lower*); *right*: 400×, magnification of square frame sections in the left; (**B**) The immunohistochemistry *PI* of brevican expression in the gliomas (5.27 ± 1.98), especially for grade III and IV astrocytoma (6.07 ± 2.30 and 8.07± 2.22), was significantly higher than that of benign brain tumors (1.78 ± 0.89). *,*P* < 0.05; **P < 0.01.

Immunohistochemical staining of 60 glioma tissue and 40 benign brain tumor samples showed that brevican was located and overexpressed in the extracellular matrix (ECM) and the cytoplasm of glioma cells, whereas the meningioma and pituitary adenoma samples were negative and weakly-positive, respectively. Diffuse positive staining for brevican was observed in glioma cells to varying degrees (Figure 1A *c -f*), compared with the benign tumor control group (Figure 1A *a, b*). The staining was especially diffuse and infiltrative for grade III and IV astrocytoma cells (Figure 1A *e, f*). The immunohistochemistry *PI* of brevican expression in the gliomas (5.27 ± 1.03) was significantly higher than that of benign brain tumors (1.78 ± 0.86, *P* < 0.05). Approximately 68.3% (41/60) of glioma samples showed positive staining (*PI* > 4.0), whereas only 22.5% (9/40) of the benign ones had positive staining, all of which were heterogeneously positive (9/20) pituitary adenoma cells (Figure 1B). In addition, the expression of brevican was not correlated with the age or sex of the patients (with *P* values of 0.10 and 0.58, respectively). However, the expression of brevican in patients with well-differentiated tumors was significantly higher than that of the patients with poorly differentiated tumors (anaplastic astrocytoma and glioblastoma) (*P*=0.005, Table 2).

**Table 2:** Brevican expression and the clinicopathological characteristics of 60 patients with malignant glioma.

**Cases**	**Brevican expression**	**Brevican expression**	***P *value**
	**positive cases (%)**	**negative cases (%)**	
Age			
≤50	25 (78.1)	7 (21.9)	0.10
>50	16 (57.1)	12 (42.9)	
Gender			
Male	22 (73.3)	8 (26.7)	0.58
Female	19 (63.3)	11 (36.7)	
Histological grade			
I	6 (40.0)	9 (60.0)	0.01
II	9 (60.0)	6 (40.0)	
III	12 (80.0)	3 (20.0)	
IV	14 (93.3)	1 (6.7)	
Degree of differentiation			
Well	15 (50.0)	15 (50.0)	0.005
Poorly	26 (86.7)	4 (13.3)	

### Brevican overexpression promoted cell adhesion and migration

**Figure 2:**
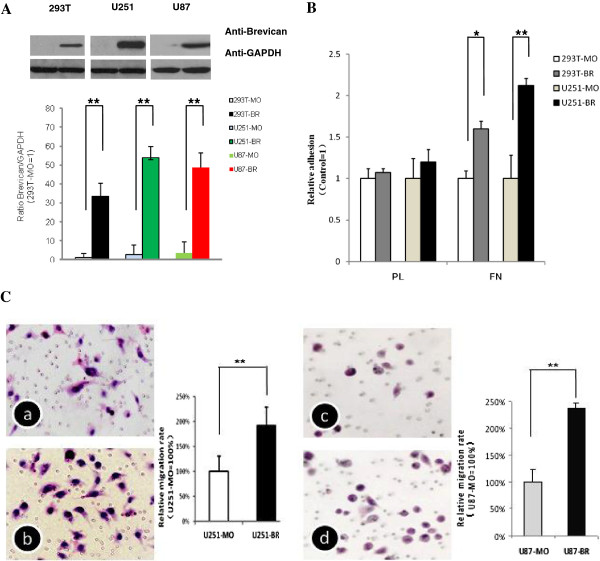
**Brevican overexpression promoted cell adhesion and migration.** (**A**) Brevican expression levels of stably transduced pMX-brevican cells were higher than those of control cells (**B**) Brevican expression enhanced fibronectin-dependent cell adhesion. PL, poly-L-lysine; FN, fibronectin. (**C**) Stably transduced U251 and U87 cells demonstrated migration through transwell inserts. Cells were stained with HE (200×), and the experiments were repeated at least three times; a: U251-MO; b: U251-BR; c: U87-MO; d: U87-BR; *MO*, the transduced cells with pMX-mock; *BR*, the transduced cells with pMX-brevican. *P < 0.05; **P < 0.01.

The glioma U251 and U87 cell lines do not express brevican in culture, probably due to the absence of microenvironment of tumor growth, i.e. specific inducing factors [8,14]. To overcome this limitation, we first generated pMX-mock- and pMX-brevican-stably- transfected 293T cells, U251 cells and U87 cells. Western blots revealed that pMX-brevican stably transduced 293T, U251 and U87 cells had much higher levels of brevican expression than control cells (Figure 2A). Therefore, these pMX-brevican stably transduced U251 cells, which were noted as “transduced U251 cells” were used for brevican overexpression experiments. Cell counting Kit-8 tests were used to ensure that the overexpression of brevican did not affect the proliferation of the transduced 293T, transduced U251, and transduced U87 cells (data not shown), as demonstrated previously by Hu et al. [15]. In cell adhesion and migration assays, the transduced cells generated traction and thereby migrated through the substrate. In this study, the results of the transwell assays showed that transfection with pMX-brevican significantly promoted the migration of U251 and U87 cells (Figure 2B). Moreover, brevican expression enhanced the fibronectin-dependent cell adhesion (Figure 2C).

### Knockdown of brevican gene inhibited cell motility abilities

**Figure 3:**
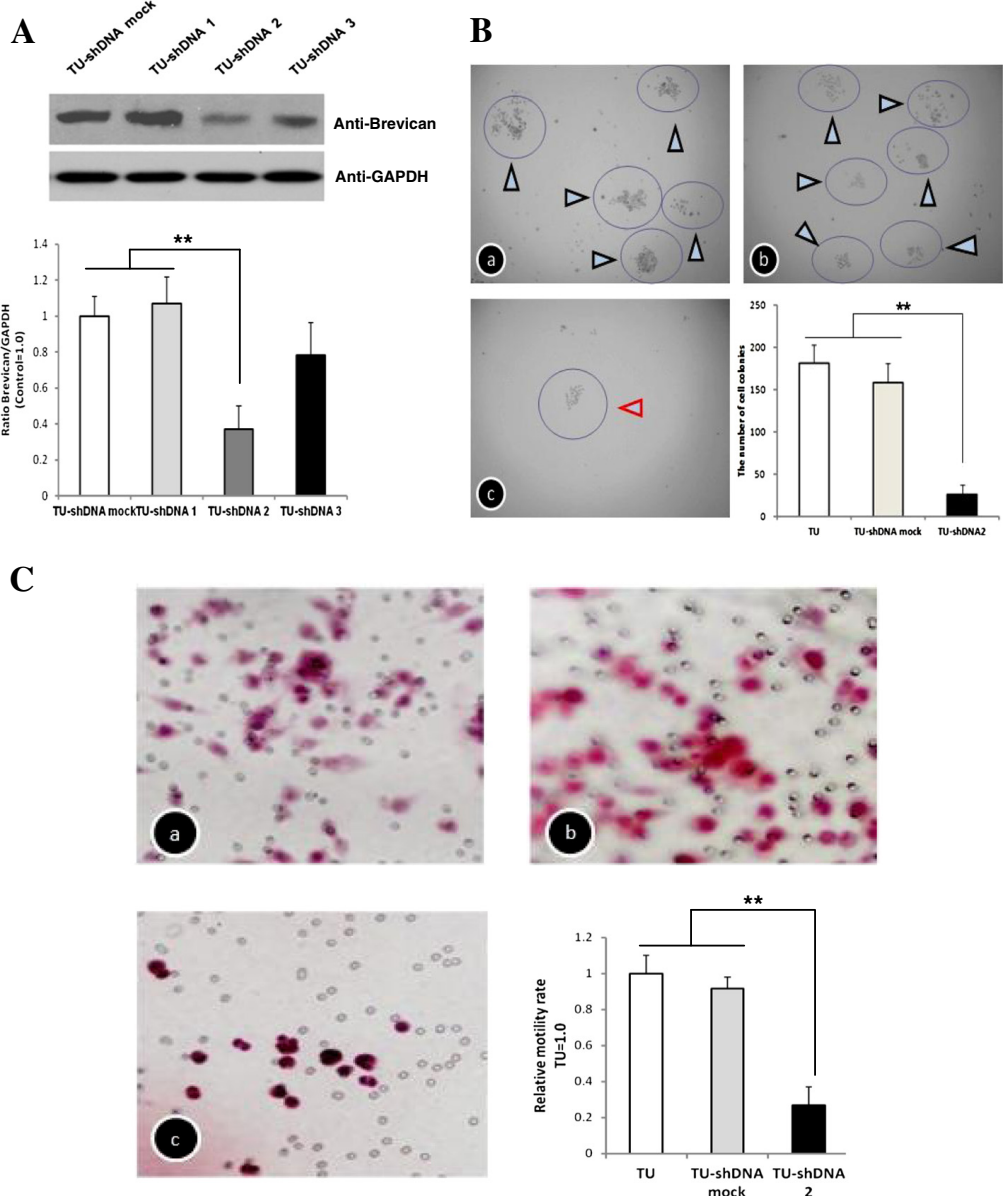
**Brevican knockdown reduced glioma cell proliferation and invasion.** (**A**) Brevican knockdown effectively down-regulated brevican expression. Western blot analysis of cell lysates demonstrated down-regulation of brevican protein in the transduced U251/brevican-shDNA 2 and the transduced U251/brevican-shDNA 3 cells by 52.3% and 23.4%, respectively. (**B**) Optical microscopic observation showed that cell colony formation was inhibited in the transduced U251/brevican-shDNA 2 cells (c), compared with the transduced U251 cells (a) and the control cells (b). (**C**) The rate of cell invasion was also reduced in the transduced U251/brevican-shDNA 2 cells (c) compared with the transduced U251 cells (a) and control cells (b). *TU*, the transduced U251 cells (a); *TU-shDNA mock*, the transduced U251/brevican-shDNA mock cells (b); *TU-shDNA2*, the transduced U251/brevican- shDNA 2 cells (c). **P < 0.01.

We knocked-down the brevican expression successfully as revealed by Western blots (Figure 3A). Cell proliferation ability was significantly reduced in the transduced U251/brevican-shDNA 2 cells compared with the transduced U251 cells and the control cells by 85.8% and 83.6% at day 12 post plating, respectively (Figure 3B). Furthermore, the invasion abilities of the transduced U251/brevican-shDNA 2 cells through the Matrigel were dramatically decreased compared with the transduced U251 cells and the control cells by 73.3% and 64.7%, respectively (*P* < 0.01; Figure 3C). These results confirmed that brevican plays an important role in glioma cell adhesion and migration.

**Figure 4:**
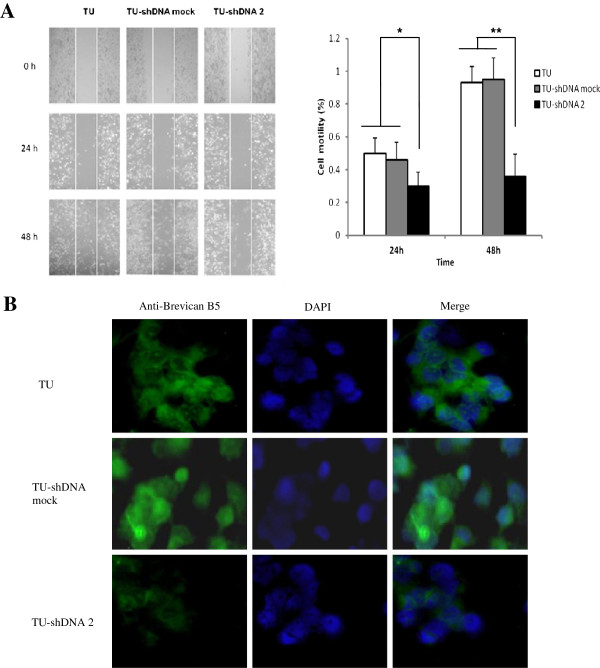
**The motility of glioma cells was decreased by brevican knockdown.** (**A**) A wound healing assay showed that the spreading ability in the transduced U251/brevican-shDNA 2 cells was significantly inhibited by brevican knockdown at 24 and 48 h. (**B**) The live transduced cells were incubated with anti-brevican antibody and subsequently processed for immunoassay. Cytoimmunofluorescence analyses also indicated the cell infiltrating ability was inhibited and brevican expression was decreased in the transduced U251/brevican-shDNA 2 cells. Brevican expression was reduced in the cytoplasm of glioma cells. *TU*, the transduced U251 cells; *TU-shDNA mock*, the transduced U251/brevican-mock cells; *TU-shDNA2*, the transduced U251/ brevican-shDNA 2 cells. *P < 0.05; **P < 0.01.

The influence of brevican on cell migration was further observed in glioma cancer cells. The wound-healing results indicated that the migration ability of the transduced U251/brevican-shDNA 2 cells was also markedly decreased (Figure 4A). As shown in Figure 4B, cytoimmunofluoresence analyses showed cell infiltrating ability was inhibited and brevican expression was decreased. The spreading distance detected in the random migration assays was significantly reduced in brevican knockdown transfectants as compared with scrambled control cells.

### Brevican knockdown inhibits tumorigenicity in vivo

**Figure 5:**
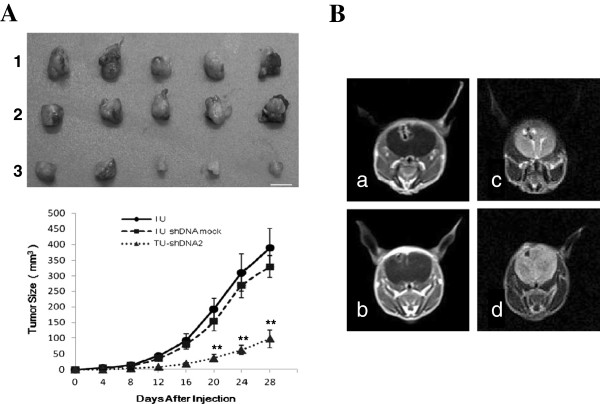
**The tumorigenicity of the transduced U251/brevican-shDNA 2 cells was reduced in nude mice.** (**A**) Photograph of subcutaneous tumors formed by the transduced U251 cells (*1*), the transduced U251/brevican-mock cells (*2*) and the transduced U251/ brevican-shDNA 2 cells (*3*) groups (n=5) at day 28. Bars are 10 mm. (**B**) The plain MRT and Gd-DTPA-enhanced MRT results for brain sections showed that the tumorigenicity of the transduced U251/brevican-shDNA 2 cells (*b, d*) was dramatically limited at day 25 (n=3), compared with the transduced U251/brevican-shDNA mock cells (*a, c*). Also, the diffusion degree of the transduced U251/brevican-mock cells (*a, c*) was more extensive than the transduced U251/brevican-shDNA 2 cells’ (*b, d*). *TU*, the transduced U251 cells; *TU-shDNA mock*, the transduced U251/brevican-mock cells; *TU-shDNA2*, transduced U251/brevican-shDNA 2 cells; *a, b*, the plain MRT results; *c, d*, Gd-DTPA-enhanced MRT results. *P < 0.05;**P < 0.01.

At the fourth week post-inoculation, the growth of tumors formed by the transduced U251/brevican-shDNA 2 cells was significantly suppressed (Figure 5A). The xenograft transplants gave rise to much smaller tumors than those from control cells (*P* < 0.05). In addition, the brains of the nude mice injected intracranially were visualized with plain and Gd-DTPA-enhanced MRT at day 25. Similar to transduced gliomas created with another glioma cell line (CNS-1) [16,17], the transduced U251/brevican-shDNA mock transfected cell-derived gliomas were invasive and exhibited cell clusters that detached from the tumor core (Figure 5B *a, c*), as well as extensive diffusion of single cells within the brain parenchyma. However, transduced U251/brevican-shDNA 2 cells produced smaller (Figure 5B *b, d*), less diffuse and less infiltrative tumors than that of the control transduced U251/brevican-shDNA mock cell tumors (volumes: 3.6 ± 1.5 mm^3^ versus 10.3 ± 2.6 mm^3^; n = 3, respectively; *P* < 0.01).

## Discussion

The ECM has an active role in regulating the activity and behavior of cells, including cell shape, differentiation, proliferation and cell death. In recent studies, the nervous ECM (NECM) was re-evaluated. To date, several studies showed that the solubility of the NECM increased in glioma, which might be related to the up-regulation of brevican [18-21].

In this study, we first explored the physiological role of brevican by investigating its spatiotemporal expression by IHC. Brevican was abundantly expressed in glioma tissues, particularly in grade III and grade IV astrocytomas, whereas brevican only expressed weakly in pituitary adenoma tumor tissue and negative in meningioma tissue. These data suggest that brevican is produced by astrocytoma cells, before being secreted and bound to the cellular cytoplasm and ECM. In the transduced brevican-expressing U251 cells, brevican was also detected on the surface of these cells using cytoimmunofluorescence. The expression of a tumor-specific brevican found in all high-grade gliomas suggested that it might play a significant role in glioma progression. Moreover, previous studies have shown that brevican is expressed at relatively low levels in normal adult brain [10,22]. Therefore, the absence or down-regulation of brevican in benign gliomas prompts its use as a differentiation marker to distinguish primary brain tumors with similar histology, but with a different pathologic course.

Here, we established an *in vitro* model to reproduce the motogenic effects of brevican. Our results indicated that brevican can promote cell adhesion and was essential for the migration of U251 cells. Brevican may interact with fibronectin (FN) to act as a motogenic signal. FN interacts with multiple cell surface receptors and plays an important role in the regulation of anchorage-dependent cell growth, cell migration, tumor development and metastasis. Cell adhesion to immobilized FN leads to the assembly of focal adhesions, which require the small GTPase Rho and then affect many cellular functions, such as cell motility, differentiation, matrix assembly, and cell cycle progression [23]. A combination of brevican cytological mechanisms and the particular composition of the neural microenvironment may underlie this unique ability of glioma to disperse in the CNS. In addition, it has been demonstrated that secreted brevican isoforms have evolved to become the predominant brevicans in the adult brain [24]. Our studies suggested that brevican overexpression in glioma is associated with cancer progression, and therefore, brevican might be a useful biomarker of glioma.

Furthermore, we also generated brevican knockdown transduced U251 cells using loss-of function techniques. We found that brevican knockdown affected cell invasion and might explain how endogenous brevican exerts its effects on cell adhesion. We confirmed that brevican can promote glioma cell adhesion [15,25]. Moreover, a distinct inhibition of the spreading and expression of brevican was observed surrounding the core of suppressed transduced U251 cells using a cytoimmunofluorescence assay. At this point we speculate that mechanisms that recycle adhesion receptors via endosomal compartments may contribute to migration. Results from our wound-healing experiments confirm this hypothesis.

Gliomas are highly invasive [26]. The ability of tumor cells to interact with the components of the NECM affects numerous cellular processes, and inappropriate expression of these matrix components has been associated with glioma invasion and growth [8,14]. One NECM component that has been implicated in glioma biology is brevican, and increasing studies have focused on brevican and mechanisms of glioma invasion in recent years [27]. Brevican’s involvement in glioma invasion may explain why many physiological processes require closely regulated degradation of the NECM [28].

In light of the effects of brevican on cell motility, we investigated the tumorigenicity of differential brevican expression *in vivo*. To date, little is known of the molecular basis that allows glioma cells to overcome the barriers that inhibit motility in the adult nervous tissue [29-31]. In this study, we found that brevican was highly overexpressed in glioma and distinctively promoted cell adhesion ability. The change of the NECM in the nervous system is critical for tumor cell aggression and invasion. To overcome the barriers to cell motility, glioma cells degrade the NECM and secrete their own matrix components. Our work demonstrated that the xenograft transplants using brevican knockdown cells gave rise to much smaller tumors, and had less diffuse and less infiltrative tumors than those of control groups. Overall, the deposition of brevican into the NECM may disrupt matrix processing and alter extracellular molecular events that modulate neural solubility.

## Conclusions

This study indicates that the expression of brevican is associated with glioma cell adhesion, motility and tumor growth. Brevican also plays an important role in glioma progression, and therefore, may be a useful marker of glioma.

## Abbreviations

FN: Fibronectin; CNS: Central nervous system; PGs: Proteoglycans; CSF: Cerebrospinal fluid; IHC: Immunohistochemical staining; HE: Hematoxylin and eosin; PI: Positive index; shDNA: Short hairpin DNA; ECM: Extracellular matrix; NECM: Nervous extracellular matrix.

## Competing interests

The authors declare that they have no competing interests.

## Authors’ contributions

RQL constructed the recombinant plasmids, detected brevican expression levels in the transduced cells, measured cell adhesion and invasion and drafted the manuscript. YCL, LG and WM collected all the tissue samples and analyzed the expression levels of brevican. CSW prepared the anti-brevican antibody and performed the immunohistochemical staining. HJW and JBD performed the *in vivo* experiments, ETW reviewed the manuscript, MY conceived and supervised the project, and reviewed the manuscript. All authors have read and approved the final manuscript.

## Pre-publication history

The pre-publication history for this paper can be accessed here:

http://www.biomedcentral.com/1471-2407/12/607/prepub
